# Anti-inflammatory and antioxidant activity of ursolic acid: a systematic review and meta-analysis

**DOI:** 10.3389/fphar.2023.1256946

**Published:** 2023-09-28

**Authors:** Man Zhao, Fengyang Wu, Zhaohong Tang, Xinyu Yang, Yanhua Liu, Fengxia Wang, Baojiang Chen

**Affiliations:** ^1^ College of Animal Science and Technology, Hebei Agricultural University, Baoding, China; ^2^ Hebei Research Institute of Microbiology Co., Ltd., Baoding, China

**Keywords:** ursolic acid, antioxidants, anti-inflammatory, *in vitro*, animals, meta-analysis

## Abstract

**Introduction:** There is currently evidence suggesting that ursolic acid may exert a favorable influence on both anti-inflammatory and antioxidant impact. Nevertheless, the anti-inflammatory and antioxidant activities of ursolic acid have not been systematically evaluated. Consequently, this study aims to conduct a systematic review and meta-analysis regarding the impact of ursolic acid on markers of inflammatory and antioxidant activity in both animal models and *in vitro* systems.

**Methods:** The search encompassed databases such as PubMed, Web of Science, Google Scholar, and ScienceDirect, up until May 2023. All eligible articles in English were included in the analysis. Standard mean difference (SMD) was pooled using a random-effects model, and the included studies underwent a thorough assessment for potential bias.

**Results:** The final review comprised 31 articles. In disease-model related studies, animal experiments have consistently shown that ursolic acid significantly reduced the levels of inflammatory parameters IL-1β, IL-6 and TNF-α in mouse tissues. In vitro studies have similarly showed that ursolic acid significantly reduced the levels of inflammatory parameters IL-1β, IL-6, IL-8 and TNF-α. Our results showed that ursolic acid could significantly elevate SOD and GSH levels, while significantly reducing MDA levels in animal tissues. The results of *in vitro* studies shown that ursolic acid significantly increased the level of GSH and decreased the level of MDA.

**Discussion:** Findings from both animal and *in vitro* studies suggest that ursolic acid decreases inflammatory cytokine levels, elevates antioxidant enzyme levels, and reduces oxidative stress levels (graphical abstract). This meta-analysis furnishes compelling evidence for the anti-inflammatory and antioxidant properties of ursolic acid.

## Graphical Abstract

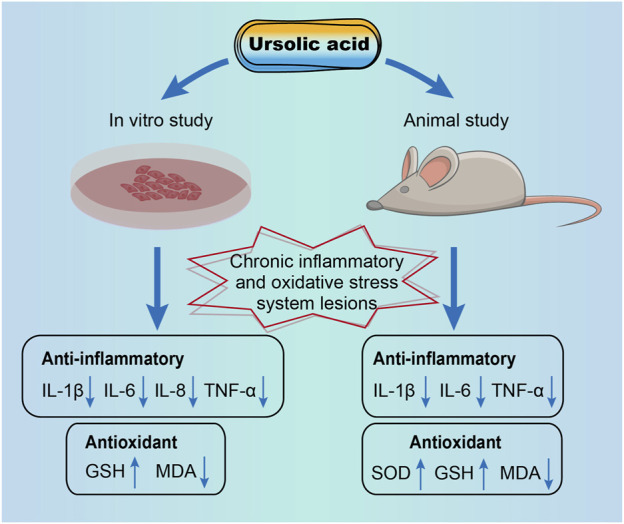

## 1 Introduction

Inflammation and oxidative stress play pivotal roles in the pathophysiology of numerous prevalent diseases, including diabetes, cardiovascular diseases, metabolic disorders, and cancer (Valko et al., 2007; Sarapultsev et al., 2015; Nakamura and Smyth, 2017; Burgos-Morón et al., 2019). In recent years, research focused on natural extracts and their bioactive constituents to mitigate inflammatory damage and ameliorate oxidative stress has gradually increased. These investigations consistently underscore the significant research and practical implications of natural extracts in mitigating the detrimental effects of inflammation and oxidative stress and reducing disease incidence (Liu et al., 2016; Moudgil and Venkatesha, 2022).

Triterpenoids are widely distributed throughout the plant kingdom, existing either as free acids or saponins in the form of sapogenins. They belong to a group of compounds long recognized for their diverse biological effects (Máñez et al., 1997). Ursolic acid, a naturally occurring pentacyclic triterpene carboxylic acid, is predominantly found in various plants, including rosemary, chasteberry, hawthorn, cranberry, and loquat leaves (Kashyap et al., 2016). Despite once being considered biologically inactive, it has garnered increasing interest in recent years due to its pharmacological potential (Kim et al., 2015; Kashyap et al., 2016; López-Hortas et al., 2018). Studies have demonstrated that ursolic acid has a broad spectrum of biological activities, including its ability to combat tumor cells and modulate lipid metabolism (Woźniak et al., 2015). Several studies have collectively affirmed the anti-inflammatory and antioxidant properties of ursolic acid (Lin et al., 2017; Li et al., 2018a). However, while several narrative review studies have explored the pharmacological effects of ursolic acid, they have not undertaken quantitative synthesis and have solely incorporated published findings (Habtemariam, 2019; Nguyen et al., 2021; Luan et al., 2022; Namdeo et al., 2023). In addition, a review of the anti-inflammatory effect of ursolic acid showed that the effects of ursolic acid on normal cells and tissues are occasionally pro-inflammatory, portraying it as a double-edged sword with both positive and negative consequences (Ikeda et al., 2008). Collectively, these studies underscore the need for a comprehensive assessment of the anti-inflammatory and antioxidant effects of ursolic acid, as well as its performance across different health conditions within the body. Systematic reviews and meta-analyses are aimed at reducing the bias of narrative reviews by identifying, appraising, and synthesizing all relevant literature, following a transparent and reproducible methodology to obtain the most reliable evidence.

Therefore, we conducted a comprehensive systematic review and meta-analysis encompassing all relevant *in vitro* and *in vivo* studies. We examined the influence of ursolic acid on the body’s health status and its role in chronic inflammatory and oxidative stress-related pathological conditions. Our objective was to ascertain whether ursolic acid possesses the capability to modulate inflammatory responses and oxidative stress.

## 2 Methods

### 2.1 Literature search strategy and selection criteria

This meta-analysis strictly followed the guidelines of the Preferred Reporting Items for Systematic Reviews and Meta-analyses (PRISMA) (Moher et al., 2015).

Three researchers independently searched PubMed, Web of Science, Google Scholar and ScienceDirect (before 6 May 2023). We restricted the language to English. The MeSH term “ursolic acid, or any name of ursolic acid, in combination with “antioxidants” or “anti-inflammatory” to identify eligible studies.

### 2.2 Inclusion and exclusion criteria

The search results from the four databases (PubMed, Web of Science, Google Scholar and ScienceDirect) were pooled in EndNote (Version X9). Duplicate publications (*n* = 1,407) were subsequently removed. For inclusion in our meta-analysis, studies had to meet the following criteria: 1) manuscripts published in English in peer-reviewed journals, 2) experimental trials and randomized control trials (RCTs), 3) studies including ursolic acid treatment and negative control groups and use of ursolic acid to treat chronic inflammatory and oxidative stress system lesions *versus* intervention control group, and 4) reporting of anti-inflammatory or antioxidant activity.

The exclusion criteria were defined as follows: 1) the major content of the supplement was not ursolic acid, 2) studies lacking data on anti-inflammatory or antioxidant activity, and 3) studies that were not of RCTs. Following these criteria, we conducted a selective screening of eligible studies for inclusion in the analysis.

### 2.3 Data extraction

The information extracted from the included studies was as follows: first author, year, country, experimental subject, sample, sample size, intervention, dosage, duration, and primary outcomes. Primary outcomes include: interleukin-1beta (IL-1β), tumor necrosis factor-alpha (TNF-α), interleukin-6 (IL-6), interleukin-8 (IL-8), interleukin-10 (IL-10), glutathione (GSH), glutathione peroxidase (GPx), catalase (CAT), superoxide dismutase (SOD), malondialdehyde (MDA). When results were available only in graphical format, data were extracted using Origin (Version 2022).

### 2.4 Study quality assessment

Two investigators (M.Z. and F.Y.W.) performed independent study quality assessment according to the criteria provided in the Consolidated Standards of Reporting Trials statement (Moher et al., 2015) and the Cochrane Collaboration’s tool for assessing risk of bias (Higgins et al., 2011). The assessment items included random sequence generation (selection bias), allocation concealment (selection bias), blinding of participants and personnel (performance bias), blinding of outcome assessment (detection bias), incomplete outcome data (attrition bias), selective reporting (reporting bias), and other bias. The divergences were resolved by the third investigator (Z.H.T.).

### 2.5 Statistical analysis

#### 2.5.1 Meta-analysis

The statistical analysis was performed with R 4.1.2 in the meta package (Balduzzi et al., 2019). The random-effects model was used to estimate the effect size and 95% confidence interval (CI) for each trait (Riley et al., 2011; Eng et al., 2014). The effect size of ursolic acid was expressed as standard mean difference (SMD). We used the *I* ^*2*^ statistic to quantitatively measure the heterogeneity in our analysis. Heterogeneity between-study variability was assessed using the *I* ^*2*^: no heterogeneity, *I* ^*2*^ ≤ 25%; low heterogeneity, 25% < *I* ^*2*^ ≤ 50%; moderate heterogeneity, 50% < *I* ^*2*^ ≤ 75%; and high heterogeneity, *I* ^*2*^ > 75% (Higgins et al., 2003).

#### 2.5.2 Meta-regression analysis

We conducted meta-regression analyses to elucidate significant heterogeneity (*p* < 0.05) or beyond a moderate level (*I* ^*2*^ > 50%) (Higgins et al., 2003). To avoid a false positive result, the regression analysis was applied only to groups with more than 10 records. Meta-regression analyses were conducted using effect sizes (SMD) for each outcome (*P*_SMD_<0.05, *I* ^*2*^ > 50%, n ≥ 10) as the dependent variable to examine heterogeneity sources of meta-analysis.

#### 2.5.3 Subgroup categorization and analysis

We conducted subgroup analyses to elucidate significant heterogeneity (*p* < 0.05) or beyond a moderate level (*I* ^*2*^ > 50%) (Higgins et al., 2003). The sub-groups were divided based on the original categories and practical implications where necessary.

#### 2.5.4 Publication bias

Publication bias was evaluated using Egger’s tests (*n* ≥ 5), for which the significance level was defined at *p* < 0.05 (Egger et al., 1997).

## 3 Results

### 3.1 Selection of studies

**FIGURE 1:**
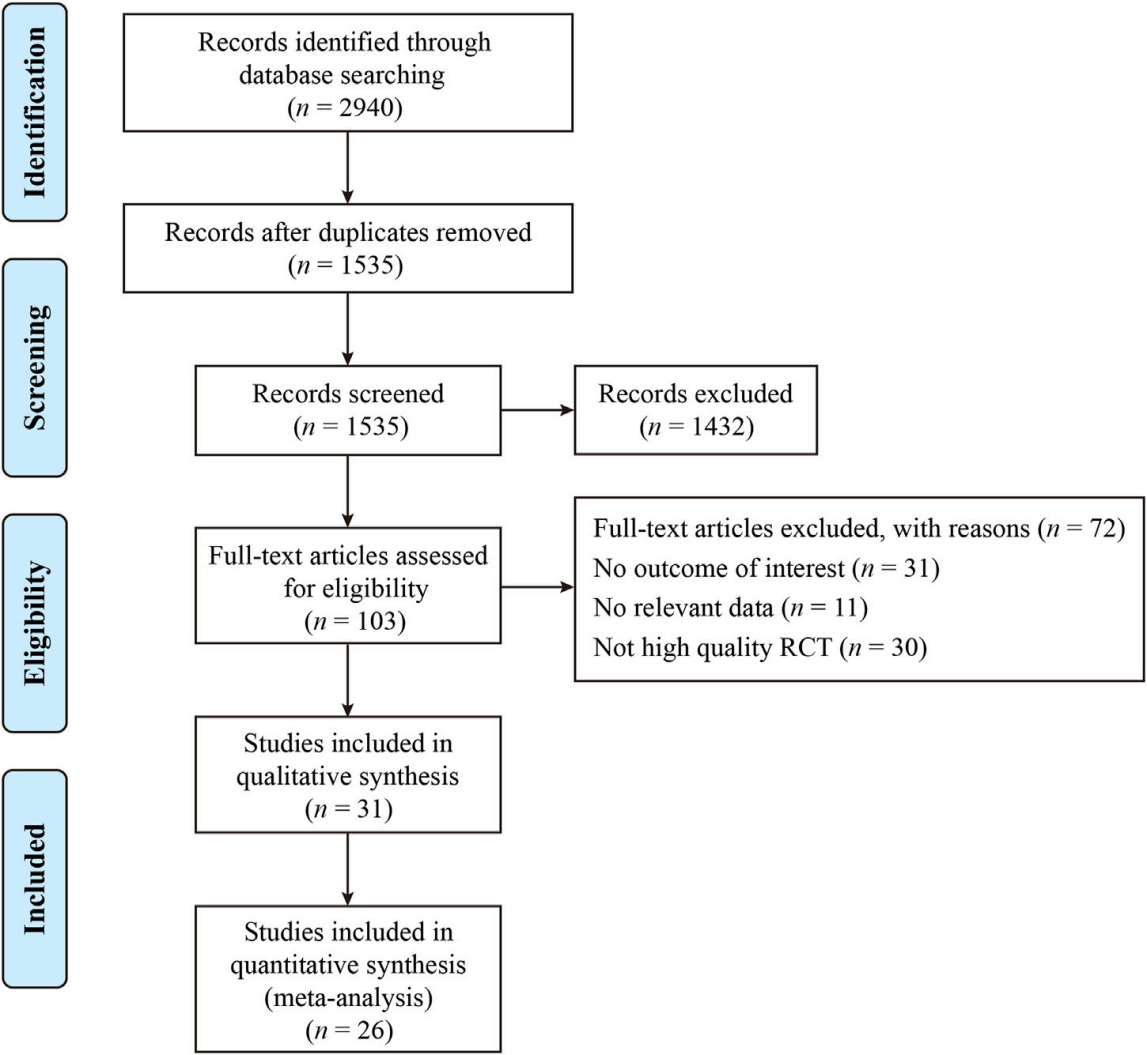
The flowchart of the search strategy and selection of eligible studies.

**FIGURE 2:**
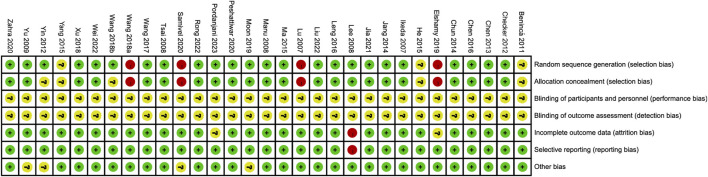
Risk of bias summary depicting authors’ judgements about each risk of bias item for each included study.

**FIGURE 3:**
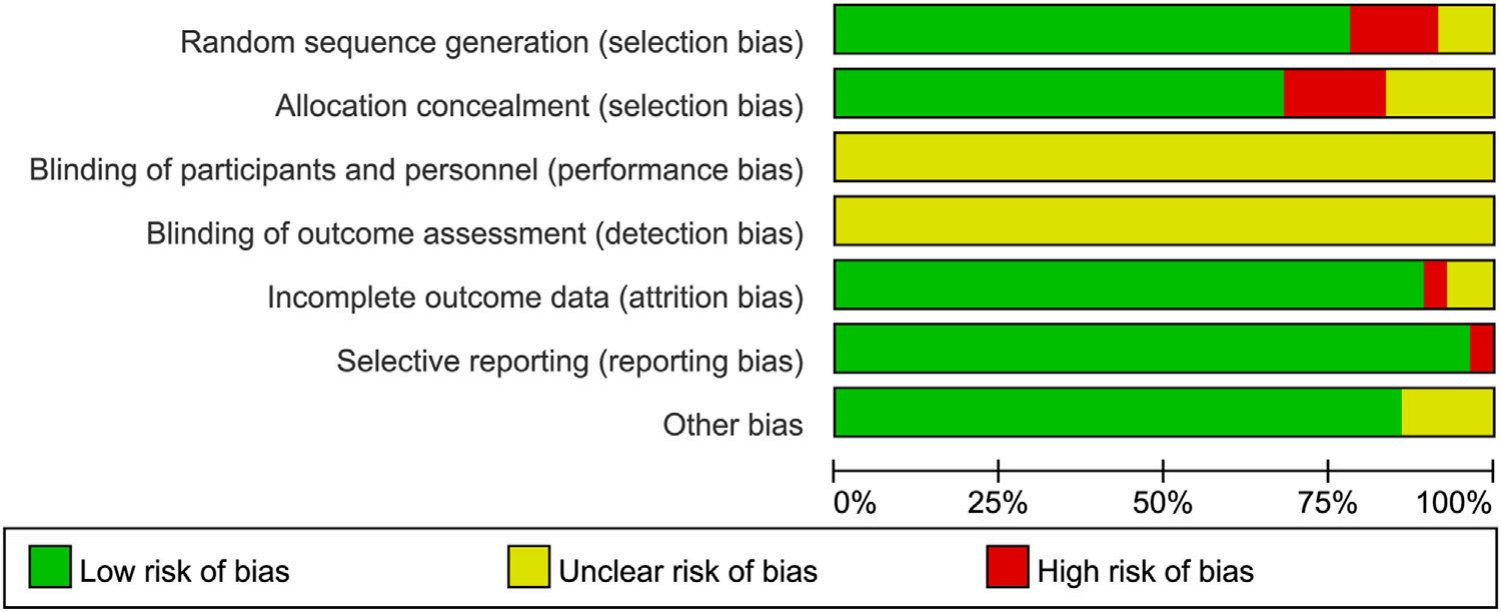
Risk of bias graph depicting review authors’ judgements about each risk of bias item presented as percentages across all included studies.

The process and results of the publication search and selection are shown in Figure 1. A total of 2,942 articles were identified, with 695 from PubMed, 1,859 from Web of Science, 94 from Google Scholar, and 294 from ScienceDirect. After removing duplicate articles, we proceeded to read the remaining 1,535 titles and abstracts. After excluding published in non-English, reviews, letters and those whose theme did not match the criteria of this study, 103 articles remained. An additional 72 articles were excluded after a full-text review based on previous protocols. Finally, 31 articles were included in this meta-analysis. The main characteristics of the 31 studies are provided in Supplementary Tables S1, S2. The bias risks for each study and overall are shown in Figure 2, Figure 3.

### 3.2 Animal studies

#### 3.2.1 The effect of use of ursolic acid to treat chronic inflammatory and oxidative stress system lesions

**FIGURE 4:**
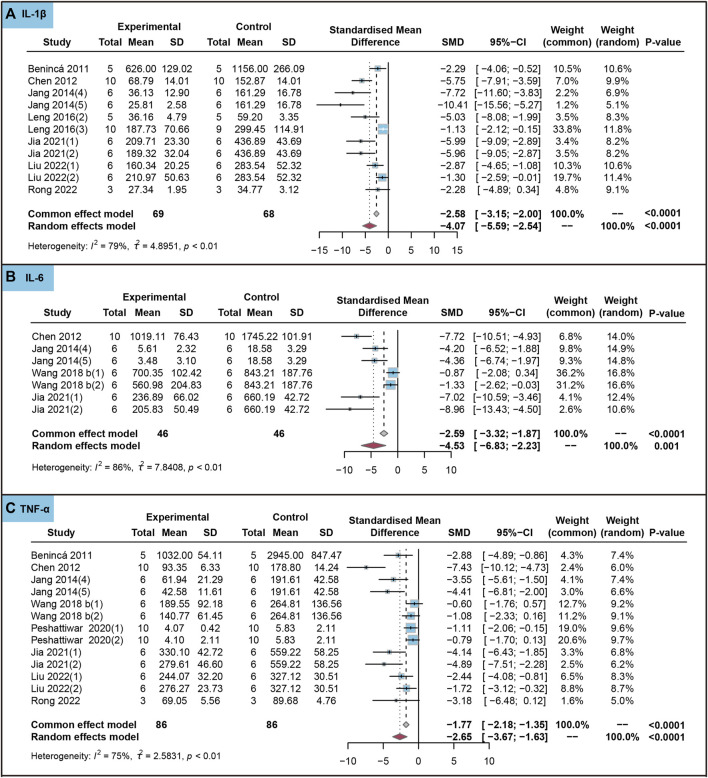
Characteristic of animal studies regarding the effect of use of ursolic acid to treat chronic inflammatory and oxidative stress system lesions on inflammatory markers. Interleukin-1beta (IL-1β) **(A)**, Interleukin-6 (IL-6) **(B)** and (Tumor necrosis factor-alpha) TNF-α **(C)**.

In animal studies, we analyzed the effect of the use of ursolic acid to treat chronic inflammatory and oxidative stress system lesions on inflammatory markers. As shown in Figure 4, ursolic acid significantly reduced the content of IL-1β (SMD = −4.07, 95%CI: −5.59 to −2.54, *P*_SMD_ < 0.0001, *I* ^*2*^ = 79%), IL-6 (SMD = −4.53, 95%CI: −6.83 to −2.23, *P*_SMD_ < 0.0001, *I* ^*2*^ = 86%) and TNF-α (SMD = −2.65, 95%CI: −3.67 to −1.63, *P*_SMD_ < 0.0001, *I* ^*2*^ = 75%).

**FIGURE 5:**
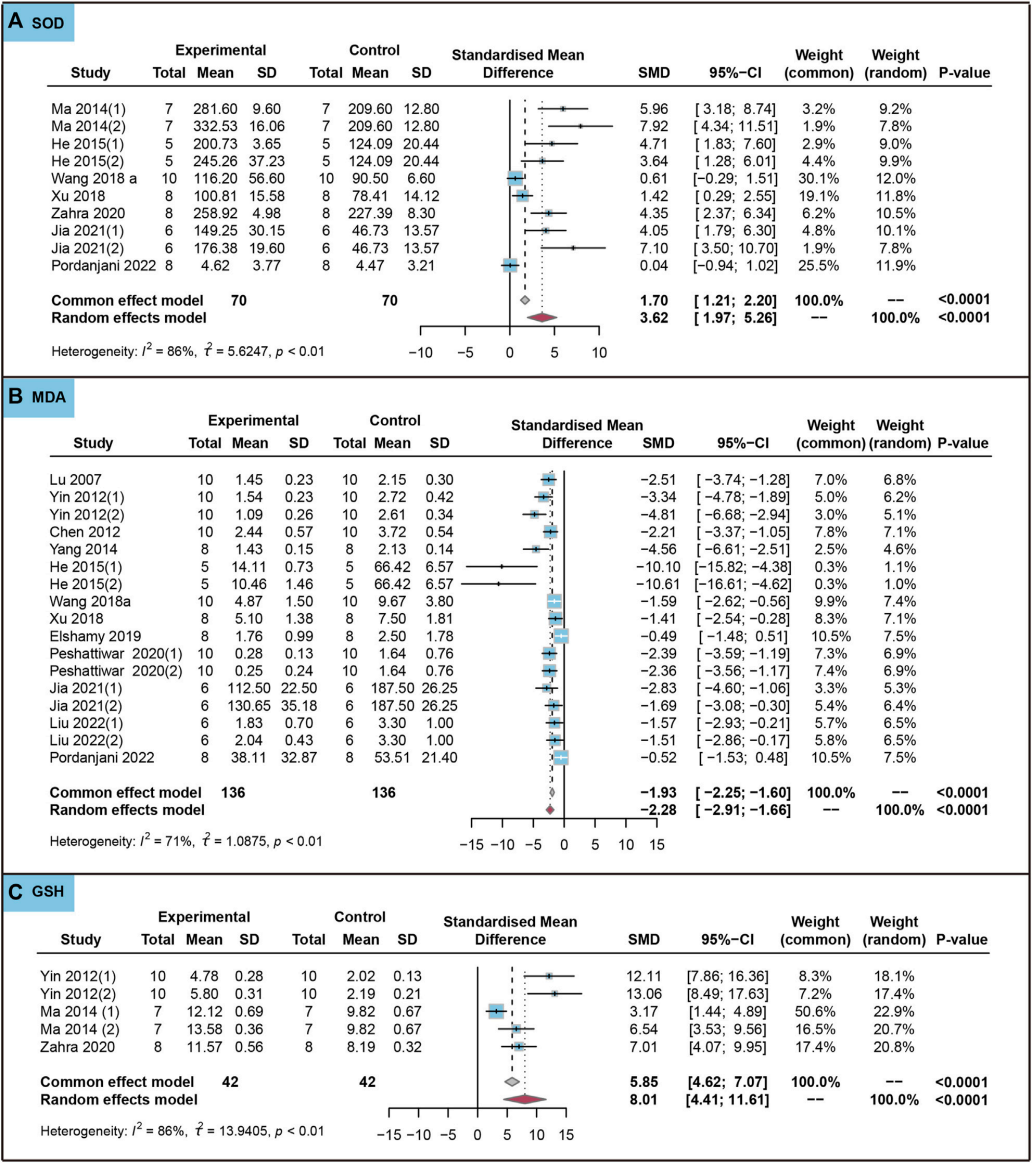
Characteristic of animal studies regarding the effect of use of ursolic acid to treat chronic inflammatory and oxidative stress system lesions on antioxidant markers. Malondialdehyde (MDA) **(A)** and Superoxide dismutase (SOD) **(B)** and Glutathione (GSH) **(C)**.

We analyzed the effects of the use of ursolic acid to treat chronic inflammatory and oxidative stress system lesions on oxidative stress markers. As shown in Figure 5, ursolic acid significantly increased the content of SOD (SMD = 3.62, 95%CI: 1.97 to 5.26, *P*_SMD_ < 0.0001, *I* ^*2*^ = 86%) and GSH (SMD = 8.01, 95%CI: 4.41 to 11.61, *P*_SMD_ < 0.0001, *I* ^*2*^ = 86%). Ursolic acid significantly reduced the content of MDA (SMD = −2.28, 95%CI: −2.91 to −1.66, *P*_SMD_ < 0.0001, *I* ^*2*^ = 71%).

We conducted meta-regression analyses, incorporating covariates such as dosage (mg/kg), duration (d) and intervention. The results showed that dosage had a significant impact on IL-1β (*p* = 0.026), while duration significantly influenced SOD (*p* = 0.002) (Table 1). However, the intervention had no significant effect on IL-1β, MDA TNF-α and SOD (*p* > 0.05). Furthermore, we conducted a sub-group analysis based on dosage categories (Low: <25 mg/kg; Middle: 25–50 mg/kg; High: > 50 mg/kg) and duration (Short (≤ 14 days); Long: >14 days). The sub-group analysis indicated that all three dosage groups of ursolic acid had significant effects on IL-1β (*P*_SMD_ < 0.05), and there was a significant difference among the three subgroups (Q = 12.05, *p* = 0.002), the low-dosage group had the best effect (SMD = −5.91, 95%CI: −8.17 to −3.65, *P*_SMD_ < 0.0001) (Table 2). Moderate heterogeneity was observed in the low-dosage group (*I* ^*2*^ = 68%, *p* < 0.05), high heterogeneity was observed in the middle-dosage group (*I* ^*2*^ = 77%, *p* < 0.05), and no heterogeneity was observed in the high-dosage group (*I* ^*2*^ = 0%, *p* = 0.51). All two duration groups of ursolic acid had significant effects on SOD (*P*_SMD_ < 0.05), but there was no significant difference between the two subgroups (*p* = 0.26). No heterogeneity was observed in the short-duration group (*I* ^*2*^ = 49%, *p* = 0.16), and high heterogeneity was observed in the long-duration group (*I* ^*2*^ = 86%, *p* < 0.05). Additionally, the results of Egger’s test indicated some evidence of publication bias in IL-1β, IL-6, MDA, SOD, TNF-α and GSH(*p* < 0.05) (Table 3).

**TABLE 1:** The summary of the meta-regression analysis.

Ursolic acid treat lesions (animal studies)				Ursolic acid treat lesions (*in vitro*)		
Dependent variable	Meta-regression parameter (*p*-value)			Dependent variable	Meta-regression parameter (*p*-value)	
	Dosage (mg/kg)	Duration (d)	Intervention		Dosage (μM)	Duration (h)
IL-1β	0.026	0.788	0.342	IL-1β	0.760	<0.0001
MDA	0.462	0.616	0.662	IL-6	0.169	0.295
TNF-α	0.857	0.339	0.637	IL-8	0.004	0.094
SOD	0.583	0.002	0.265	TNF-α	0.945	0.395

Abbreviations: IL-1β, interleukin-1beta; MDA, malondialdehyde; TNF-α, tumor necrosis factor-alpha; SOD, superoxide dismutase; IL-6, interleukin-6; IL-8, interleukin-8.

**TABLE 2:** The summary of the sub-group analysis.

—			Random effects model				Test of heterogeneity		Subgroup differences	
			n	Random effect	95% CI	*p*-value	Heterogeneity *I* ^*2*^	*p*-value	Q	*p*-value
Ursolic acid treat lesions (animal studies)										
** **IL-1β	All trials	—	69	−4.07	[-5.59; −2.54]	<0.0001	79 (%)	<0.01	—	—
	Dosage	Low (<25 mg/kg)	34	−5.91	[-8.17; −3.65]	<0.0001	68	0.01	12.05	0.002
		Middle (25–50 mg/kg)	26	−3.24	[-5.46; −1.02]	0.0043	77	<0.01		
		High (>50 mg/kg)	9	−1.49	[-2.65; −0.33]	0.0116	0	0.51		
** **SOD	All trials	—	70	3.62	[1.97; 5.26]	<0.0001	86	<0.01	—	—
	Duration	Short (≤14 days)	12	5.24	[2.32; 8.15]	0.0004	49	0.16	1.28	0.26
		Long (>14 days)	58	3.24	[1.40; 5.09]	0.0006	86	<0.01		
Ursolic acid treat lesions (*in vitro*)										
** **IL-1β	All trials	—	39	−5.89	[−8.24; −3.54]	<0.0001	60	<0.01		
	Duration	Short (<20 h)	12	−1.85	[−3.23; −0.48]	0.008	45	0.14	18.56	<0.0001
		Middle (20–40 h)	18	−6.94	[−9.43; −4.45]	<0.0001	0	0.56		
		Long (>40 h)	9	−10.28	[−15.80; −4.76]	<0.0001	0	0.88		
** **IL-8	All trials	—	42	−6.43	[−8.91; −3.94]	<0.0001	53	<0.01	—	—
	Dosage	Low (<10 μM)	15	−4.26	[−7.23; −1.29]	0.0049	48	0.10	6.09	0.048
		Middle (10–19 μM)	6	−5.78	[−9.67; −1.88]	0.0036	0	0.79		
		High (>19 μM)	21	−14.00	[−21.15; −6.86]	0.0001	38	0.14		

Abbreviations: IL-1β, interleukin-1beta; SOD, superoxide dismutase; IL-8, interleukin-8.

**TABLE 3:** The summary of Egger’s tests.

Parameter	Egger’s test					
	Ursolic acid (*in vitro*)		Ursolic acid treat lesions (animal studies)		Ursolic acid treat lesions (*in vitro*)	
	t	*p*-value	t	*p*-value	t	*p*-value
IL-1β	7.83	0.080	−6.85	<0.0001	−13.5	<0.0001
IL-6	0.19	0.869	−8.25	0.0004	−7.45	<0.0001
MDA			−5.79	< 0.0001	−2.93	0.022
SOD			10.74	< 0.0001	0.28	0.789
TNF-α			−6.76	< 0.0001	−4.62	0.0003
GSH			13.84	0.0008	2.95	0.098
GPx	—	—	—	—	−0.54	0.604
CAT	—	—	—	—	1.06	0.326
IL-10	—	—	—	—	120.58	0.005
IL-8	—	—	—	—	−70.81	<0.0001

Abbreviations: IL-1β, interleukin-1beta; IL-6, interleukin-6; MDA, malondialdehyde; SOD, su-peroxide dismutase; TNF-α, tumor necrosis factor-alpha; GPx, glutathione peroxidase; CAT, catalase; IL-10, interleukin-10; GSH, glutathione; IL-8, interleukin-8.

### 3.3 *In vitro* studies

#### 3.3.1 The effect of ursolic acid on anti-inflammatory and antioxidant parameters

**FIGURE 6:**
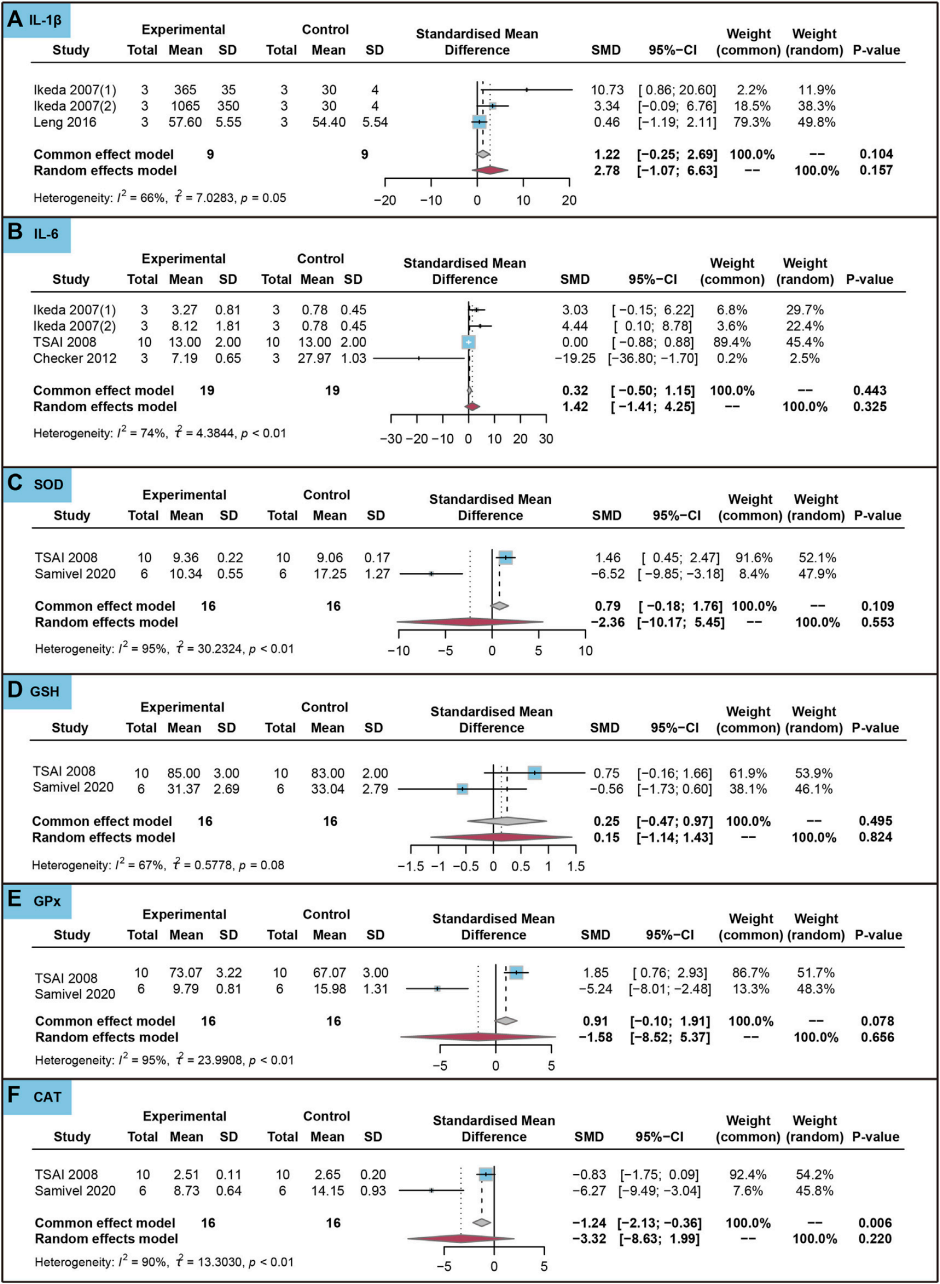
Characteristic of *in vitro* studies regarding the effect of ursolic acid on inflammatory and antioxidant markers. Interleukin-1beta (IL-1β) **(A)**, Interleukin-6 (IL-6) **(B)**, Superoxide dismutase (SOD) **(C)**, Glutathione (GSH) **(D)**, Glutathione peroxidase (GPx) **(E)** and Catalase (CAT) **(F)**.

As shown in Figure 6, ursolic acid did not significantly affect IL-1β, IL-6, SOD, GSH, GPx and CAT in healthy cells (*P*_SMD_ > 0.05). The Egger’s test results indicated no evidence of publication bias in IL-1β and IL-6 (*p* > 0.05).

#### 3.3.2 The effect of use of ursolic acid to treat chronic inflammatory and oxidative stress system lesions

**FIGURE 7:**
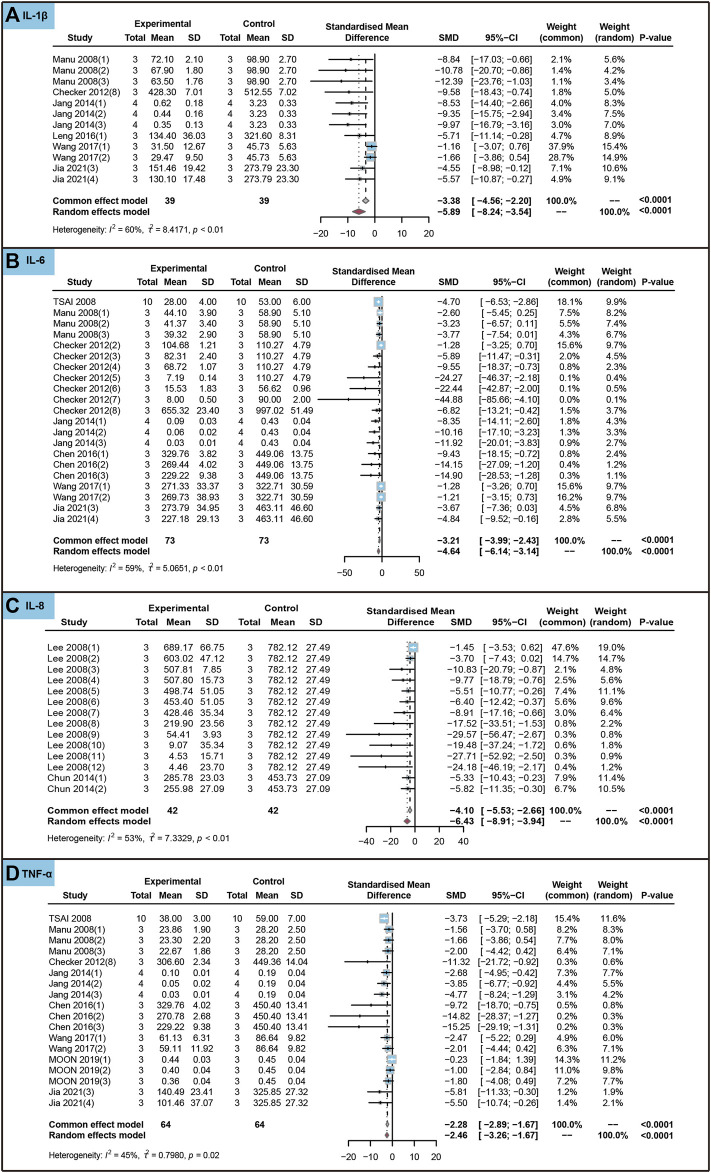
Characteristic of *in vitro* studies regarding the effect of use of ursolic acid to treat chronic inflammatory and oxidative stress system lesions on inflammatory markers. Interleukin-1beta (IL-1β) **(A)**, Interleukin-6 (IL-6) **(B)**, Interleukin-8 (IL-8) **(C)** and Tumor necrosis factor-alpha (TNF-α) **(D)**.

We conducted an analysis to evaluate the impact of ursolic acid in treating chronic inflammatory and oxidative stress system lesions on inflammatory markers within cells. As shown in Figure 7, ursolic acid significantly reduced the content of IL-1β (SMD = −5.89, 95%CI: −8.24 to −3.54, *P*_SMD_ < 0.0001, *I* ^*2*^ = 60%), IL-6 (SMD = −4.64, 95%CI: −6.14 to −3.14, *P*_SMD_ < 0.0001, *I* ^*2*^ = 59%), IL-8 (SMD = −6.43, 95%CI: −8.91 to −3.94, *P*_SMD_ < 0.0001, *I* ^*2*^ = 53%) and TNF-α (SMD = −2.46, 95%CI: −3.26 to −1.67, *P*_SMD_ < 0.0001, *I* ^*2*^ = 45%).

**FIGURE 8:**
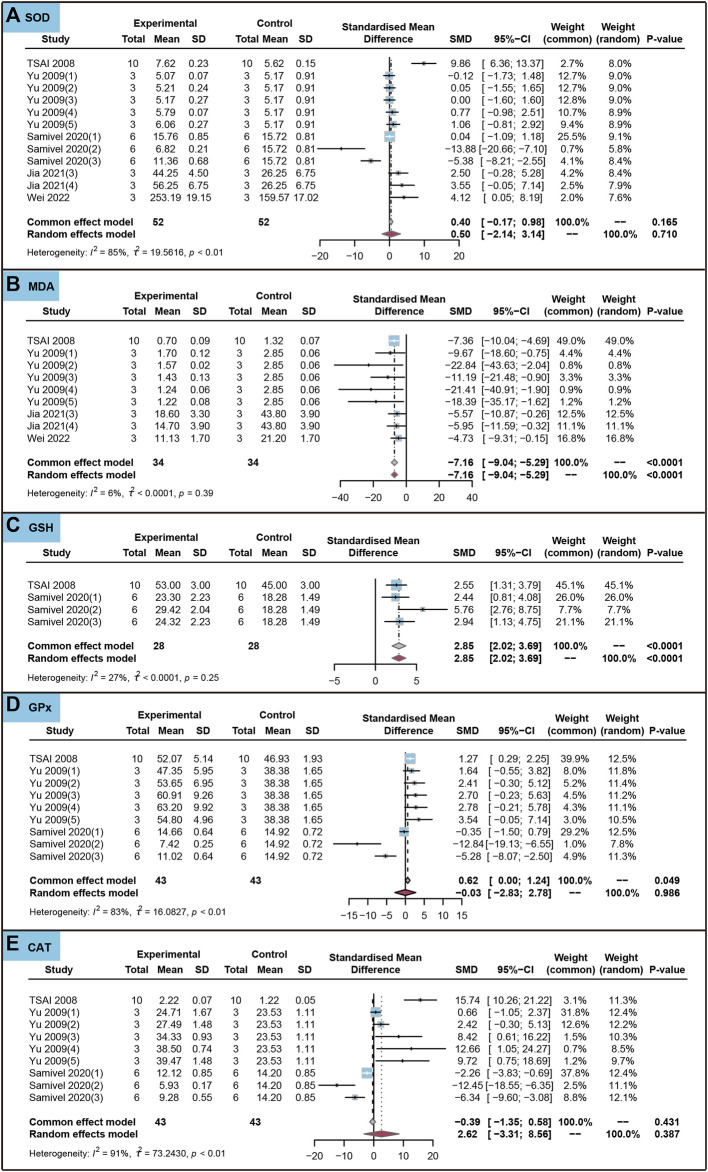
Characteristic of *in vitro* studies regarding the effect of use of ursolic acid to treat chronic inflammatory and oxidative stress system lesions on antioxidant markers. Superoxide dismutase (SOD) **(A)**, Malondialdehyde (MDA) **(B)**, Glutathione (GSH) **(C)**, Glutathione peroxidase (GPx) **(D)** and Catalase (CAT) **(E)**.

We analyzed the effects of the use of ursolic acid to treat chronic inflammatory and oxidative stress system lesions on oxidative stress markers in cells. As shown in Figure 8, ursolic acid did not significantly affect withers SOD, GPx and CAT (*P*_SMD_ > 0.05). Ursolic acid significantly reduced the content of MDA (SMD = −7.16, 95%CI: −9.04 to −5.29, *P*_SMD_ < 0.0001, *I* ^*2*^ = 6%). Ursolic acid significantly increased the content of GSH (SMD = 2.85, 95%CI: 2.02 to 3.69, *P*_SMD_ < 0.0001, *I* ^*2*^ = 27%).

We performed meta-regression analyses, including two covariates: dosage (μM) and duration (h). The results revealed that dosage had a significant effect on IL-8 (*p* = 0.004) but no significant effect on IL-1β, IL-6 and TNF-α (*p* > 0.05). On the other hand, duration had a significant effect on IL-1β (*p* < 0.0001) but no significant effect on IL-6, IL-8 and TNF-α (*p* > 0.05) (Table 1). Subsequently, we conducted sub-group analyses for these two covariates, with dosage categorized as Low: <10 μM, Middle: 10–19 μM, High: >19 μM; and duration as Short: <20 h, Middle: 20–40 h, Long: >40 h). Sub-group analysis indicated that all three duration groups of ursolic acid had significant effects on IL-1β (*P*_SMD_<0.05), Notably, there was a significant difference among the three subgroups (Q = 18.56, *p* < 0.0001), with the long-duration group demonstrating the most favorable effect (SMD = −10.28, 95%CI: −15.80 to −4.76, *P*_SMD_ < 0.0001) (Table 2). No heterogeneity was observed in three duration groups (Short: *I* ^*2*^ = 45%, Middle: *I* ^*2*^ = 0%, Long: *I* ^*2*^ = 0%; *p* > 0.05). Furthermore, the sub-group analysis indicated that all three dosage groups of ursolic acid had significant effects on IL-8 (*P*_SMD_<0.05), Similarly, there was a significant difference among the three subgroups (Q = 6.09, *p* = 0.048), with the high dosage group had the best effect (SMD = −14.00, 95%CI: −21.15 to −6.86, *P*_SMD_ = 0.0001) (Table 2). No heterogeneity was observed in three dosage groups (Short: *I* ^*2*^ = 48%, Middle: *I* ^*2*^ = 0%, Long: *I* ^*2*^ = 38%; *p* > 0.05). Finally, the results of Egger’s test indicated some evidence of publication bias in IL-1β, IL-6, IL-8, IL-10, MDA and TNF-α (*p* < 0.05) (Table 3), while no evidence of publication bias was found in SOD, GSH, GPx and CAT (*p* > 0.05).

## 4 Discussion

This meta-analysis aims to comprehensively evaluate the impact of ursolic acid on enhancing anti-inflammatory and antioxidant functions while identifying potential influencing factors on effect size. The findings hold substantial potential for optimizing the application of ursolic acid, which carries significant implications for preventing and treating diseases related to inflammatory and oxidative stress systems. Distinguished from previous studies, our systematic review encompassed multiple databases and, as far as possible, conducted quantitative syntheses. To the best of our knowledge, this is currently a rare comprehensive meta-analysis of this issue. This systematic review and meta-analysis of 31 trials suggests that ursolic acid has a significant beneficial effect in the treatment of inflammatory and oxidative stress system diseases.

In the context of an inflammatory response, there is a noticeable increase in the expression and release of pro-inflammatory cytokines. The excessive production of these inflammatory factors stands as a primary driver of tissue damage. Among the key pro-inflammatory cytokines, IL-1 and TNF-α occupy prominent roles. TNF-α, as the initiating cytokine in the inflammatory process, triggers cascading effects that stimulate the upregulation of other inflammatory factors such as IL-1 and IL-6, thereby amplifying the overall inflammatory response (Dinarello, 2005; Hovhannisyan et al., 2011; Bent et al., 2018; Pandolfi et al., 2020; Bernhard et al., 2021; van Loo and Bertrand, 2023). In disease-model-related studies, animal experiments have consistently shown that ursolic acid significantly reduced the levels of inflammatory parameters IL-1β, IL-6 and TNF-α in mouse tissues. *In vitro* studies have similarly showed that ursolic acid significantly reduced the levels of inflammatory parameters IL-1β, IL-6, IL-8 and TNF-α. These findings collectively support the notion that ursolic acid significantly inhibits inflammatory processes. In addition, it is plausible to assert that cells and animals experiencing inflammatory responses may benefit more from ursolic acid compared to healthy states. This observation might also be attributed to the frequent inclusion of ursolic acid in studies aimed at preventing and treating inflammatory diseases. In addition, the high consistency of results from animal and cellular studies further supports the anti-inflammatory activity of ursolic acid.

Antioxidant defense systems exist in most organisms, effectively scavenging free radicals and ROS. The redox dynamic balance is determined by the balance between the production of free radicals and ROS and their elimination by various antioxidants. Key enzymatic antioxidants in animals include SOD, GSH, GPx, and CAT (Fang et al., 2002). When there is an increased production of oxygen radicals or oxidizing substances in the body beyond the antioxidant defense mechanism of the cells, it results in cellular oxidative stress (Sarapultsev et al., 2015). MDA a byproduct of lipid peroxidation, serves as an indicator of cellular damage and a biomarker for oxidative stress severity (Tangvarasittichai, 2015). Several natural extracts, including vitamin C, vitamin E, flavonoids and polyphenols have demonstrated their ability to bolster the body’s innate defense system and maintain redox homeostasis (Rubió et al., 2013). Our results showed that ursolic acid could significantly elevate SOD and GSH levels, while significantly reducing MDA levels in animal tissues. The results of *in vitro* studies shown that ursolic acid significantly increased the level of GSH and decreased the level of MDA. Hence, it is plausible to consider ursolic acid as a non-enzymatic antioxidant capable of fortifying cellular and organismal antioxidant defenses, thereby mitigating oxidative stress.

Interestingly, a previous study showed that ursolic acid increased IL-1β levels in healthy mice in a concentration- and time-dependent manner (Ikeda et al., 2007a). A high dosage of ursolic acid was associated with increased IL-1β production in healthy mice (Ikeda et al., 2007b). Notably, the impact of ursolic acid on IL-1β appeared to show opposite results in healthy *versus* diseased mice. While the exact reason for this discrepancy remains unclear, the aforementioned study suggests that the dose and duration of ursolic acid treatment could be decisive factors in its effects. Therefore, through regression analysis and subgroup analysis, we explored whether these two factors influence the effect of ursolic acid treatment and attempted to identify the source of heterogeneity. Ultimately, we identified dosage as a crucial factor affecting ursolic acid’s ability to reduce IL-1 levels in animals. Lower doses (< 25 mg/kg) yielded a more potent therapeutic effect, whereas no therapeutic effect was found in the high-dosage group (>50 mg/kg). However, high heterogeneity remained in the low and intermediate dose groups, indicating the existence of other factors that may modulate ursolic acid’s anti-inflammatory effects. In conclusion, ursolic acid possesses a robust anti-inflammatory effect, with lower doses being more efficacy, suggesting that ursolic acid has a complex mechanism of action and that pro-inflammatory effects may arise at higher doses.

In an investigation into the *in vitro* study, duration significantly affected IL-1β levels, with longer durations (> 40 h) being able to reduce IL-1β levels more substantially. Notably, we did not observe heterogeneity in any of the three subgroups, suggesting that varying durations may be the primary source of heterogeneity. Consequently, it appears that a longer duration is a critical factor contributing to the anti-inflammatory effects of ursolic acid *in vitro*. Interestingly, we also noted inconsistencies between factors affecting *in vivo* and *in vitro* studies. These disparities may be attributed to factors such as the bioavailability of ursolic acid, which could help elucidate these differences. In our *in vitro* investigations, we observed that dose was the main factor influencing the effect of ursolic acid on IL-8 production, where high doses (> 19 μM) were able to produce greater therapeutic effects, and the low heterogeneity in the three subgroups suggests that different doses were a source of heterogeneity across these studies. Additionally, our observations indicated that ursolic acid significantly reduces SOD levels in animal studies, whereas no consistent effect was observed in *in vitro* studies. This inconsistency may be attributed to differences between *in vitro* and *in vivo* biological modeling systems.

The precise mechanisms underlying the effects of ursolic acid supplementation on markers of inflammation and oxidative stress remain elusive. However, we have suggested some potential mechanisms for ursolic acid and markers of inflammation and oxidative stress in our meta-analysis. Major molecular targets of Inflammatory diseases include pro-inflammatory cytokines and their receptors, nuclear factor kappa B (NF-κB), c-Jun-N-terminal kinases (JNK) and mitogen-activated protein kinases (MAPK) (Gautam and Jachak, 2009). Ursolic acid can suppress NF-κB activation by inhibiting IκB kinase and p65 phosphorylation (Shishodia et al., 2003). Notably, NF-κB regulates the expression of genes associated with proinflammatory cytokines (Sun, 2017; Yu et al., 2020). Thus, the anti-inflammatory effect of ursolic acid may be through the inhibition of NF-κB to reduce inflammatory markers. Another pathway may involve ursolic acid exerting its anti-inflammatory effects by inhibiting the MAPK signaling pathway (Ma et al., 2014). Regarding its antioxidant function, ursolic acid may operate by scavenging free radicals (Shih et al., 2004; Li et al., 2018b). In addition, ursolic acid has been shown to inhibit oxidative stress through the liver kinase B1 (LKB1)-activated protein kinase (AMPK) signaling pathway (Yang et al., 2015).

Ursolic acid demonstrates the potential to reduce the marker levels of inflammation and oxidative stress, making it a valuable candidate for both preventive measures and adjunctive treatments in cases of chronic inflammation and oxidative stress-related damage. Given that chronic inflammation and oxidative stress tend to escalate with age and are implicated in the onset of numerous age-related diseases, the versatility of ursolic acid makes it particularly promising, especially for elderly individuals. Ursolic acid has been proposed as a therapeutic option for addressing conditions such as rheumatism, arthritis, and metabolic disorders (Kim et al., 2015; Nguyen et al., 2021). Moreover, considering that older adults often contend with multiple medical conditions necessitating polypharmacy, ursolic acid’s low potential for drug-drug interactions renders it even more advantageous. Consequently, older adults grappling with chronic inflammatory and oxidative stress-related issues stand to benefit significantly from the potential therapeutic effects of ursolic acid.

While we aimed for a comprehensive review in this study, it is essential to acknowledge its limitations: 1) We did not assess clinical outcomes and there are no ongoing randomized controlled trials assessing the effect of ursolic acid on clinical outcomes. 2) Despite our efforts to mitigate heterogeneity through regression and subgroup analyses, substantial heterogeneity persisted in certain parameters due to the limited number of included studies. Exclusion of individual studies did not significantly alter this heterogeneity. 3) In the majority of experimental studies, there was a lack of information regarding blinding procedures for participants, personnel, and outcome assessment. This omission introduces uncertainty regarding the risk of bias, particularly in terms of performance and detection bias. The strength of this study lies in its comprehensive systematic review of all relevant animal and *in vitro* studies. Ursolic acid, while promising, remains an unproven natural compound. Nonetheless, our findings underscore the potential role of ursolic acid in anti-inflammatory processes and in enhancing antioxidant defense mechanisms.

## 5 Conclusion

This meta-analysis suggests that ursolic acid may indeed lower levels of inflammatory cytokines, increase antioxidant enzyme levels, and reduce oxidative stress levels. Notably, it appears that cells and animals with existing inflammatory responses may benefit more from ursolic acid compared to healthy states. This study addresses the controversy over the effects of ursolic acid on inflammatory and oxidative stress processes and provides a basis for future applications of ursolic acid. To advance this field of research, it is crucial to conduct future studies with heightened methodological precision, larger sample sizes, and increased consistency in parameters such as dosage/concentration and route of administration. By doing so, we can gain a clearer understanding of the strength of the relationship between ursolic acid and inflammation and oxidative stress. This can ultimately pave the way for more definitive large-scale randomized controlled trials, both for treatment and prevention purposes, which are clearly warranted.

## Data Availability

The original contributions presented in the study are included in the article/Supplementary Material, further inquiries can be directed to the corresponding author.

## References

[B1] Balduzzi S. Rücker G. Schwarzer G. (2019). How to perform a meta-analysis with R: A practical tutorial. Evid. Based Ment. Health 22 (4), 153–160. 10.1136/ebmental-2019-300117 31563865 PMC10231495

[B2] Bent R. Moll L. Grabbe S. Bros M. (2018). Interleukin-1 beta-A friend or foe in malignancies? Int. J. Mol. Sci. 19 (8), 2155. 10.3390/ijms19082155 30042333 PMC6121377

[B3] Bernhard S. Hug S. Stratmann A. E. P. Erber M. Vidoni L. Knapp C. L. (2021). Interleukin 8 elicits rapid physiological changes in neutrophils that are altered by inflammatory conditions. J. Innate Immun. 13 (4), 225–241. 10.1159/000514885 33857948 PMC8460987

[B4] Burgos-Morón E. Abad-Jiménez Z. Marañón A. M. Iannantuoni F. Escribano-López I. López-Domènech S. (2019). Relationship between oxidative stress, ER stress, and inflammation in type 2 diabetes: The battle continues. J. Clin. Med. 8 (9), 1385. 10.3390/jcm8091385 31487953 PMC6780404

[B5] Dinarello C. A. (2005). Blocking IL-1 in systemic inflammation. J. Exp. Med. 201 (9), 1355–1359. 10.1084/jem.20050640 15867089 PMC2213199

[B6] Egger M. Davey Smith G. Schneider M. Minder C. (1997). Bias in meta-analysis detected by a simple, graphical test. Bmj 315 (7109), 629–634. 10.1136/bmj.315.7109.629 9310563 PMC2127453

[B7] Eng C. Kramer C. K. Zinman B. Retnakaran R. (2014). Glucagon-like peptide-1 receptor agonist and basal insulin combination treatment for the management of type 2 diabetes: A systematic review and meta-analysis. Lancet 384 (9961), 2228–2234. 10.1016/s0140-6736(14)61335-0 25220191

[B8] Fang Y. Z. Yang S. Wu G. (2002). Free radicals, antioxidants, and nutrition. Nutrition 18 (10), 872–879. 10.1016/s0899-9007(02)00916-4 12361782

[B9] Gautam R. Jachak S. M. (2009). Recent developments in anti-inflammatory natural products. Med. Res. Rev. 29 (5), 767–820. 10.1002/med.20156 19378317

[B10] Habtemariam S. (2019). Antioxidant and anti-inflammatory mechanisms of neuroprotection by ursolic acid: Addressing brain injury, cerebral ischemia, cognition deficit, anxiety, and depression. Oxid. Med. Cell. Longev. 2019, 8512048. 10.1155/2019/8512048 31223427 PMC6541953

[B11] Higgins J. P. Altman D. G. Gøtzsche P. C. Jüni P. Moher D. Oxman A. D. (2011). The Cochrane Collaboration's tool for assessing risk of bias in randomised trials. Bmj 343, d5928. 10.1136/bmj.d5928 22008217 PMC3196245

[B12] Higgins J. P. Thompson S. G. Deeks J. J. Altman D. G. (2003). Measuring inconsistency in meta-analyses. Bmj 327 (7414), 557–560. 10.1136/bmj.327.7414.557 12958120 PMC192859

[B13] Hovhannisyan Z. Treatman J. Littman D. R. Mayer L. (2011). Characterization of interleukin-17-producing regulatory T cells in inflamed intestinal mucosa from patients with inflammatory bowel diseases. Gastroenterology 140 (3), 957–965. 10.1053/j.gastro.2010.12.002 21147109 PMC3049831

[B14] Ikeda Y. Murakami A. Fujimura Y. Tachibana H. Yamada K. Masuda D. (2007a). Aggregated ursolic acid, a natural triterpenoid, induces IL-1beta release from murine peritoneal macrophages: Role of CD36. J. Immunol. 178 (8), 4854–4864. 10.4049/jimmunol.178.8.4854 17404266

[B15] Ikeda Y. Murakami A. Fujimura Y. Tachibana H. Yamada K. Masuda D. (2007b). Aggregated ursolic acid, a natural triterpenoid, induces IL-1beta release from murine peritoneal macrophages: Role of CD36. J. Immunol. 178 (8), 4854–4864. 10.4049/jimmunol.178.8.4854 17404266

[B16] Ikeda Y. Murakami A. Ohigashi H. (2008). Ursolic acid: An anti- and pro-inflammatory triterpenoid. Mol. Nutr. Food Res. 52 (1), 26–42. 10.1002/mnfr.200700389 18203131

[B17] Kashyap D. Tuli H. S. Sharma A. K. (2016). Ursolic acid (ua): A metabolite with promising therapeutic potential. Life Sci. 146, 201–213. 10.1016/j.lfs.2016.01.017 26775565

[B18] Kim M. H. Kim J. N. Han S. N. Kim H. K. (2015). Ursolic acid isolated from guava leaves inhibits inflammatory mediators and reactive oxygen species in LPS-stimulated macrophages. Immunopharmacol. Immunotoxicol. 37 (3), 228–235. 10.3109/08923973.2015.1021355 25753845

[B19] Li J. Li N. Yan S. Liu M. Sun B. Lu Y. (2018a). Ursolic acid alleviates inflammation and against diabetes-induced nephropathy through TLR4-mediated inflammatory pathway. Mol. Med. Rep. 18 (5), 4675–4681. 10.3892/mmr.2018.9429 30221655

[B20] Li Q. Zhao W. Zeng X. Hao Z. (2018b). Ursolic acid attenuates atherosclerosis in ApoE(-/-) mice: Role of LOX-1 mediated by ROS/NF-κB pathway. Molecules 23 (5), 1101. 10.3390/molecules23051101 29735887 PMC6100321

[B21] Lin L. Yin Y. Hou G. Han D. Kang J. Wang Q. (2017). Ursolic acid attenuates cigarette smoke-induced emphysema in rats by regulating PERK and Nrf2 pathways. Pulm. Pharmacol. Ther. 44, 111–121. 10.1016/j.pupt.2017.03.014 28347799

[B22] Liu J. Li X. Lin J. Li Y. Wang T. Jiang Q. (2016). Sarcandra glabra (caoshanhu) protects mesenchymal stem cells from oxidative stress: A bioevaluation and mechanistic chemistry. BMC Complement. Altern. Med. 16 (1), 423. 10.1186/s12906-016-1383-7 27793132 PMC5084467

[B23] López-Hortas L. Pérez-Larrán P. González-Muñoz M. J. Falqué E. Domínguez H. (2018). Recent developments on the extraction and application of ursolic acid. A review. Food Res. Int. 103, 130–149. 10.1016/j.foodres.2017.10.028 29389599

[B24] Luan M. Wang H. Wang J. Zhang X. Zhao F. Liu Z. (2022). Advances in anti-inflammatory activity, mechanism and therapeutic application of ursolic acid. Mini Rev. Med. Chem. 22 (3), 422–436. 10.2174/1389557521666210913113522 34517797

[B25] Ma J.-Q. Ding J. Zhang L. Liu C.-M. J. E. t. (2014). Ursolic acid protects mouse liver against CCl4-induced oxidative stress and inflammation by the MAPK/NF-κB pathway. Environ. Toxicol. Pharmacol. 37 (3), 975–983. 10.1016/j.etap.2014.03.011 24727148

[B26] Máñez S. Recio M. C. Giner R. M. Ríos J. L. (1997). Effect of selected triterpenoids on chronic dermal inflammation. Eur. J. Pharmacol. 334 (1), 103–105. 10.1016/s0014-2999(97)01187-4 9346335

[B27] Moher D. Shamseer L. Clarke M. Ghersi D. Liberati A. Petticrew M. (2015). Preferred reporting items for systematic review and meta-analysis protocols (PRISMA-P) 2015 statement. Syst. Rev. 4 (1), 1. 10.1186/2046-4053-4-1 25554246 PMC4320440

[B28] Moudgil K. D. Venkatesha S. H. (2022). The anti-inflammatory and immunomodulatory activities of natural products to control autoimmune inflammation. Int. J. Mol. Sci. 24 (1), 95. 10.3390/ijms24010095 36613560 PMC9820125

[B29] Nakamura K. Smyth M. J. (2017). Targeting cancer-related inflammation in the era of immunotherapy. Immunol. Cell. Biol. 95 (4), 325–332. 10.1038/icb.2016.126 27999432

[B30] Namdeo P. Gidwani B. Tiwari S. Jain V. Joshi V. Shukla S. S. (2023). Therapeutic potential and novel formulations of ursolic acid and its derivatives: An updated review. J. Sci. Food Agric. 103 (9), 4275–4292. 10.1002/jsfa.12423 36597140

[B31] Nguyen H. N. Ullevig S. L. Short J. D. Wang L. Ahn Y. J. Asmis R. (2021). Ursolic acid and related analogues: Triterpenoids with broad health benefits. Antioxidants (Basel) 10 (8), 1161. 10.3390/antiox10081161 34439409 PMC8388988

[B32] Pandolfi F. Franza L. Carusi V. Altamura S. Andriollo G. Nucera E. (2020). Interleukin-6 in rheumatoid arthritis. Int. J. Mol. Sci. 21 (15), 5238. 10.3390/ijms21155238 32718086 PMC7432115

[B33] Riley R. D. Higgins J. P. Deeks J. J. (2011). Interpretation of random effects meta-analyses. Bmj 342, d549. 10.1136/bmj.d549 21310794

[B34] Rubió L. Motilva M. J. Romero M. P. (2013). Recent advances in biologically active compounds in herbs and spices: A review of the most effective antioxidant and anti-inflammatory active principles. Crit. Rev. Food Sci. Nutr. 53 (9), 943–953. 10.1080/10408398.2011.574802 23768186

[B35] Sarapultsev P. A. Chupakhin O. N. Medvedeva S. U. Mukhlynina E. A. Brilliant S. A. Sidorova L. P. (2015). The impact of immunomodulator compound from the group of substituted thiadiazines on the course of stress reaction. Int. Immunopharmacol. 25 (2), 440–449. 10.1016/j.intimp.2015.02.024 25737199

[B36] Shih Y. H. Chein Y. C. Wang J. Y. Fu Y. S. (2004). Ursolic acid protects hippocampal neurons against kainate-induced excitotoxicity in rats. Neurosci. Lett. 362 (2), 136–140. 10.1016/j.neulet.2004.03.011 15193771

[B37] Shishodia S. Majumdar S. Banerjee S. Aggarwal B. B. (2003). Ursolic acid inhibits nuclear factor-kappaB activation induced by carcinogenic agents through suppression of IkappaBalpha kinase and p65 phosphorylation: Correlation with down-regulation of cyclooxygenase 2, matrix metalloproteinase 9, and cyclin D1. Cancer Res. 63 (15), 4375–4383. 12907607

[B38] Sun S. C. (2017). The non-canonical NF-κB pathway in immunity and inflammation. Nat. Rev. Immunol. 17 (9), 545–558. 10.1038/nri.2017.52 28580957 PMC5753586

[B39] Tangvarasittichai S. (2015). Oxidative stress, insulin resistance, dyslipidemia and type 2 diabetes mellitus. World J. Diabetes 6 (3), 456–480. 10.4239/wjd.v6.i3.456 25897356 PMC4398902

[B40] Valko M. Leibfritz D. Moncol J. Cronin M. T. Mazur M. Telser J. (2007). Free radicals and antioxidants in normal physiological functions and human disease. Int. J. Biochem. Cell. Biol. 39 (1), 44–84. 10.1016/j.biocel.2006.07.001 16978905

[B41] van Loo G. Bertrand M. J. M. (2023). Death by TNF: A road to inflammation. Nat. Rev. Immunol. 23 (5), 289–303. 10.1038/s41577-022-00792-3 36380021 PMC9665039

[B42] Woźniak Ł. Skąpska S. Marszałek K. (2015). Ursolic acid-A pentacyclic triterpenoid with a wide spectrum of pharmacological activities. Molecules 20 (11), 20614–20641. 10.3390/molecules201119721 26610440 PMC6332387

[B43] Yang Y. Zhao Z. Liu Y. Kang X. Zhang H. Meng M. J. J. o. g. (2015). Suppression of oxidative stress and improvement of liver functions in mice by ursolic acid via LKB 1‐AMP‐activated protein kinase signaling. J. Gastroenterol. Hepatol. 30 (3), 609–618. 10.1111/jgh.12723 25168399

[B44] Yu H. Lin L. Zhang Z. Zhang H. Hu H. (2020). Targeting NF-κB pathway for the therapy of diseases: Mechanism and clinical study. Signal Transduct. Target Ther. 5 (1), 209. 10.1038/s41392-020-00312-6 32958760 PMC7506548

