# Supplementary material for: Anti-inflammatory and antioxidant activity of ursolic acid: a systematic review and meta-analysis
Source: Front Pharmacol. 2023 Sep 28;14:1256946. doi: 10.3389/fphar.2023.1256946 (PMC10568483; doi:10.3389/fphar.2023.1256946)
Supplement: Supplementary file 1 [file DataSheet1.docx]

Supplementary Material

Anti-inflammatory and antioxidant activity of ursolic acid: A systematic review and meta-analysis

**Man Zhao, Fengyang Wu, Zhaohong Tang, Xinyu Yang, Yanhua Liu, Fengxia Wang, Baojiang Chen ***

*** Correspondence:** Baojiang Chen: chenbaojiang@vip.sina.com

# Supplementary Tables

**Table S1** Characteristics of the included animal studies in the meta-analysis

| **Study** | **Country** | **Breed** | **Sample** | **N** | **Intervention** | **Dosage** | **Durationd** | **Lesion studied** | **Parameters** |
| --- | --- | --- | --- | --- | --- | --- | --- | --- | --- |
| Benincá et al. (2011) [1] | Brazil | Mice | Pleural fluid | 5 | Intraperitoneal injection | 25 mg/kg | 4 h | Inflammation induction by carrageenan (1%) | TNF-α；IL-1β |
| Chen et al. (2012) [2] | China | Mice | Serum | 10 | Intraperitoneal injection | 10 mg/kg | 24 h | Lipopolysaccharide (LPS)-acute lung injury | TNF-α；MDA；IL-1β; IL-6 |
| Elshamy et al. (2019) [3] | Egypt | Rats | Stomach | 8 | Oral | 100 mg/kg | 24 h | Ethanol-challenged | MDA |
| He et al. (2015) [4] | China | Rats | Serum | 5 | Intraperitoneal injections | 20; 40 mg/kg | 4 weeks | Liver fibrosis model | MDA；SOD |
| Jang et al. (2014) [5] | Korea | Mice | Colon | 6 | Oral | 10; 20 mg/kg | 3 days | Colitis model | TNF-α；IL-6；IL-1β |
| Jia et al. (2021) [6] | China | Rats | Kidney tissue | 6 | Intragastric administration | 20; 40 mg/ kg | 4 weeks | Kidney injury model | TNF-α；MDA；IL-6；IL-1β；SOD |
| Leng et al. (2016) [7] | China | Mice | Serum | 5 | Intraperitoneal injection | 50 mg/kg | 16 h | Lipopolysaccharide-induced inflammation model | IL-1β |
| Leng et al. (2016) [7] | China | Mice | Serum | 9; 10 | Intraperitoneal injection | 50 mg/kg | 11 weeks | Atherosclerosis model | IL-1β |
| Liu et al. (2022) [8] | China | Rats | Hippocampal tissues | 6 | Intragastric administration | 20; 100 mg/kg | 7 days | Neuroinflammation model | TNF-α；MDA；IL-1β |
| Lu et al. (2007) [9] | China | Mice | Brain tissues | 10 | Oral | 10 mg/kg | 2 weeks | Neurotoxicity model | MDA |
| Ma et al. (2014) [10] | China | Mice | Plasma | 7 | Oral | 25; 50 mg/kg | 6 weeks | Hepatotoxicity and fibrosis model | SOD; GSH; CAT |
| Peshattiwar et al. (2020) [11] | India | Rats | Brain tissues | 10 | Oral | 5; 10 mg/kg | 30 days | Degeneration of dopaminergic neurons | TNF-α；MDA |
| Pordanjani et al. (2022) [12] | Iran | Rats | Blood | 8 | Oral | 500 mg/kg | 8 weeks | Type 2 diabetes model | MDA；SOD |
| Rong et al. (2022) [13] | China | Mice | Colon tissue | 3 | Intragastric administration | 200 mg/kg | 20 days | Spinal injury | TNF-α；IL-1β |
| Wang et al. (2018)a [14] | China | Rats | Cardiac tissue | 10 | Intragastric administration | 35 mg/kg | 8 weeks | Diabetes mellitus model | MDA；SOD |
| Wang et al. (2018)b [15] | China | Rats | Cortex | 6 | Intragastric administration | 10; 20 mg/kg | 47 h | Cerebral ischemia and reperfusion injury | TNF-α；IL-6 |
| Xu et al. (2018) [16] | China | Rats | Serum | 8 | Intragastric administration | 35 mg/kg | 3 weeks | Diabetes mellitus model | MDA；SOD |
| Yang et al. (2014) [17] | China | Mice | Liver | 8 | Intraperitoneal injection | 50 mg/kg | 2 weeks | Liver injury model | MDA |
| Yin et al. (2012) [18] | China | Mice | Renal homogenates | 10 | Oral | 250 mg/kg | 4; 8 weeks | Lipid oxidation model | MDA |
| Zahra et al. (2020) [19] | India | Mice | Brains | 8 | Oral | 25 mg/kg | 6 weeks | Rotenone-Induced Parkinsonism | SOD; GSH |

Abbreviations: IL-1β, interleukin-1beta; IL-6, interleukin-6; MDA, malondialdehyde; SOD, superoxide dismutase; TNF-α, tumor necrosis factor-alpha; GPx, glutathione peroxidase; CAT, catalase; IL-10, interleukin-10; GSH, glutathione; IL-8, interleukin-8.

**Table S2** Characteristics of the included in vitro studies in the meta-analysis

| **Study** | **Country** | **Breed** | **N** | **Dosage** | **Durationd** | **Lesion studied** | **Parameters** |
| --- | --- | --- | --- | --- | --- | --- | --- |
| Ikeda et al. (2007) [20] | Japan | Macrophag | 3 | 4; 20 μM | 12 h | Health | IL-1β；IL-6 |
| Leng et al. (2016) [7] | China | Macrophage | 3 | 10 μM | 6 h | Health | IL-1β |
| Samivel et al. (2020) [21] | Saudi Arabia | Human skin dermal fibroblasts | 6 | 20 μM | 24 h | Health | GSH；GPx；CAT；SOD |
| TSAI et al. (2008) [22] | China | PC12 cells: rat adrenal gland pheochromocytoma cell line | 10 | 40 μM | 48 h | Health | IL-6；GSH；GPx；CAT；SOD |
| Checker et al. (2012) [23] | India | Lymphocytes | 3 | 5 μM | 4 h | Health | IL-6 |
| Checker et al. (2012) [23] | India | Lymphocytes | 3 | 0.25; 0.5; 1; 5 μM | 4 h | Lymphocyte proliferation | IL-6 |
| Checker et al. (2012) [23] | India | CD4+T cells | 3 | 5 μM | 4 h | Lymphocyte proliferation | IL-6 |
| Checker et al. (2012) [23] | India | Macrophages | 3 | 5 μM | 4 h | Lipopolysaccharide induced inflammation model | IL-6; IL-1β; TNF-α |
| Chen et al. (2016) [24] | China | Murine preosteoblastic calvarial cells MC3T3E1 | 3 | 0.01; 0.1; 1 μM | 24 h | Glucocorticoid-induced osteoporosis model | TNF-α；IL-6 |
| Chun et al. (2014) [25] | Korea | The human intestinal epithelial cell line COLO 205 | 3 | 10; 50 μM | 24 h | Stimulated with TNF-α (10 ng/ml) | IL-8 |
| Jang et al. (2014) [5] | Korea | Macrophages | 4 | 5; 10; 20 μM | 20 h | Lipopolysaccharide induced inflammation model | TNF-α；IL-6；IL-1β |
| Jia et al. (2021) [6] | China | HK-2 cells: renal tubular epithelium | 3 | 2.5; 5 μM | 24 h | Kidney damage induced by crystals of calcium oxalate monohydrate | TNF-α；IL-6；IL-1β；MDA；SOD |
| Lee et al. (2008) [26] | China | Human lung epithelial cells (A-549) | 3 | 0.5; 1.0; 2.0; 3.9; 7.8; 15.6; 31.3; 62.5; 125; 250; 500; 1000 μM | 30 min | Lipopolysaccharide induced inflammation model | IL-8 |
| Leng et al. (2016) [7] | China | Macrophage | 3 | 10 μM | 6 h | Lipopolysaccharide induced inflammation model | TNF-α；IL-6；IL-1β |
| Manu et al. (2008) [27] | India | B16F-10 melanoma cells | 3 | 10; 25; 50 μM | 48 h | Melanoma | TNF-α；IL-6；IL-1β |
| MOON et al. (2019) [28] | Korea | Human mast cell line HMc-1 | 3 | 0.002; 0.02; 0.2 μg/ml | 8 h | Phorbol myristate acetate and calcium ionophore stimulation | TNF-α |
| Samivel et al. (2020) [21] | Saudi Arabia | Human skin dermal fibroblasts | 6 | 10; 20; 40 μM | 24; 48 h | Ultraviolet B irradiation | GSH；GPx；CAT；SOD |
| TSAI et al. (2008) [22] | China | PC12 cells: rat adrenal gland pheochromocytoma cell  line | 10 | 40 μM | 48 h | Exposure of hydrogen peroxide | TNF-α；IL-6；MDA；GSH；GPx；CAT；SOD |
| Wang et al. (2017) [29] | Singapore | HaCaT cells: immortalized human epidermal keratinocyte | 3 | 10; 15 μM | 16 h | 20 Gy gamma radiation induced damage | TNF-α；IL-6；IL-1β |
| Wei et al. (2022) [30] | China | Type II alveolar epithelial cell line (human, A549) | 3 | 20 μg/ml | 48 h | Influenza A virus infection | MDA；SOD |
| Yu et al. (2009) [31] | Korea | HEI-OC1 auditory cells | 3 | 0.05; 0.1; 0.5; 1; 2 μg/ml | 25 h | Exposure of hydrogen peroxide | MDA；GPx；CAT；SOD |

Abbreviations: IL-1β, interleukin-1beta; IL-6, interleukin-6; MDA, malondialdehyde; SOD, superoxide dismutase; TNF-α, tumor necrosis factor-alpha; GPx, glutathione peroxidase; CAT, catalase; IL-10, interleukin-10; GSH, glutathione; IL-8, interleukin-8.

**Articles included in this meta-analysis:**

1. Beninca, J.P.; Dalmarco, J.B.; Pizzolatti, M.G.; Froede, T.S. Analysis of the anti-inflammatory properties of Rosmarinus officinalis L. in mice. Food chemistry 2011, 124, 468-475, doi:10.1016/j.foodchem.2010.06.056.

2. Chen, X.; Wan, Y.; Zhou, T.; Li, J.; Wei, Y. Ursolic acid attenuates lipopolysaccharide-induced acute lung injury in a mouse model. IMMUNOTHERAPY 2013, 5, 39-47, doi:10.2217/IMT.12.144.

3. Elshamy, A.I.; Farrag, A.-R.H.; Mohamed, S.H.; Ali, N.A.; Mohamed, T.A.; Menshawy, M.M.; Zaglool, A.W.; Efferth, T.; Hegazy, M.-E.F. Gastroprotective effects of ursolic acid isolated from Ochrosia elliptica on ethanol-induced gastric ulcer in rats. MEDICINAL CHEMISTRY RESEARCH 2020, 29, 113-125, doi:10.1007/s00044-019-02465-8.

4. He, W.; Shi, F.; Zhou, Z.-W.; Li, B.; Zhang, K.; Zhang, X.; Ouyang, C.; Zhou, S.-F.; Zhu, X.J.D.D., Development; Therapy. A bioinformatic and mechanistic study elicits the antifibrotic effect of ursolic acid through the attenuation of oxidative stress with the involvement of ERK, PI3K/Akt, and p38 MAPK signaling pathways in human hepatic stellate cells and rat liver. 2015, 9, 3989.

5. Jang, S.-E.; Jeong, J.-J.; Hyam, S.R.; Han, M.J.; Kim, D.-H. Ursolic Acid Isolated from the Seed of Cornus officinalis Ameliorates Colitis in Mice by Inhibiting the Binding of Lipopolysaccharide to Toll-like Receptor 4 on Macrophages. Journal of agricultural and food chemistry 2014, 62, 9711-9721, doi:10.1021/jf501487v.

6. Jia, Z.; Li, W.; Bian, P.; Yang, L.; Liu, H.; Pan, D.; Dou, Z.J.B. Ursolic acid treats renal tubular epithelial cell damage induced by calcium oxalate monohydrate via inhibiting oxidative stress and inflammation. 2021, 12, 5450-5461.

7. Leng, S.; Iwanowycz, S.; Saaoud, F.; Wang, J.; Wang, Y.; Sergin, I.; Razani, B.; Fan, D. Ursolic acid enhances macrophage autophagy and attenuates atherogenesis[S]. Journal of lipid research 2016, 57, 1006-1016, doi:https://doi.org/10.1194/jlr.M065888.

8. Liu, K.-m.; Huang, Y.; Wan, P.-p.; Lu, Y.-h.; Zhou, N.; Li, J.-j.; Yu, C.-y.; Chou, J.-j.; Zhang, L.; Zhang, C.J.F.i.P. Ursolic acid protects neurons in temporal lobe epilepsy and cognitive impairment by repressing inflammation and oxidation. 2022, 13.

9. Lu, J.; Zheng, Y.L.; Wu, D.M.; Luo, L.; Sun, D.X.; Shan, Q. Ursolic acid ameliorates cognition deficits and attenuates oxidative damage in the brain of senescent mice induced by D-galactose. Biochemical pharmacology 2007, 74, 1078-1090, doi:10.1016/j.bcp.2007.07.007.

10. Ma, J.Q.; Ding, J.; Zhang, L.; Liu, C.M. Protective effects of ursolic acid in an experimental model of liver fibrosis through Nrf2/ARE pathway. Clinics and research in hepatology and gastroenterology 2015, 39, 188-197, doi:10.1016/j.clinre.2014.09.007.

11. Peshattiwar, V.; Muke, S.; Kaikini, A.; Bagle, S.; Dighe, V.; Sathaye, S. Mechanistic evaluation of Ursolic acid against rotenone induced Parkinson’s disease– emphasizing the role of mitochondrial biogenesis. Brain research bulletin 2020, 160, 150-161, doi:https://doi.org/10.1016/j.brainresbull.2020.03.003.

12. Pordanjani, M.K.; Banitalebi, E.; Roghani, M.; Hemmati, R. Ursolic acid enhances the effect of exercise training on vascular aging by reducing oxidative stress in aged type 2 diabetic rats. Food science & nutrition 2023, 11, 696-708, doi:10.1002/fsn3.3105.

13. Rong, Z.-J.; Cai, H.-H.; Wang, H.; Liu, G.-H.; Zhang, Z.-W.; Chen, M.; Huang, Y.-L. Ursolic Acid Ameliorates Spinal Cord Injury in Mice by Regulating Gut Microbiota and Metabolic Changes. Frontiers in cellular neuroscience 2022, 16, doi:10.3389/fncel.2022.872935.

14. Wang, X.-t.; Gong, Y.; Zhou, B.; Yang, J.-j.; Cheng, Y.; Zhao, J.-g.; Qi, M.-y.J.B.; Pharmacotherapy. Ursolic acid ameliorates oxidative stress, inflammation and fibrosis in diabetic cardiomyopathy rats. 2018, 97, 1461-1467.

15. Wang, Y.; Li, L.; Deng, S.; Liu, F.; He, Z. Ursolic Acid Ameliorates Inflammation in Cerebral Ischemia and Reperfusion Injury Possibly via High Mobility Group Box 1/Toll-Like Receptor 4/NFκB Pathway. Frontiers in neurology 2018, 9, 253, doi:10.3389/fneur.2018.00253.

16. Xu, H.L.; Wang, X.T.; Cheng, Y.; Zhao, J.G.; Zhou, Y.J.; Yang, J.J.; Qi, M.Y. Ursolic acid improves diabetic nephropathy via suppression of oxidative stress and inflammation in streptozotocin-induced rats. Biomedicine & pharmacotherapy = Biomedecine & pharmacotherapie 2018, 105, 915-921, doi:10.1016/j.biopha.2018.06.055.

17. Yang, Y.; Zhao, Z.; Liu, Y.; Kang, X.; Zhang, H.; Meng, M.J.J.o.g.; hepatology. Suppression of oxidative stress and improvement of liver functions in mice by ursolic acid via LKB 1‐AMP‐activated protein kinase signaling. 2015, 30, 609-618.

18. Yin, M.-C.; Lin, M.-C.; Mong, M.-C.; Lin, C.-Y. Bioavailability, Distribution, and Antioxidative Effects of Selected Triterpenes in Mice. Journal of agricultural and food chemistry 2012, 60, 7697-7701, doi:10.1021/jf302529x.

19. Zahra, W.; Rai, S.N.; Birla, H.; Singh, S.S.; Rathore, A.S.; Dilnashin, H.; Singh, R.; Keswani, C.; Singh, R.K.; Singh, S.P. Neuroprotection of Rotenone-Induced Parkinsonism by Ursolic Acid in PD Mouse Model. CNS & neurological disorders drug targets 2020, 19, 527-540, doi:10.2174/1871527319666200812224457.

20. Ikeda, Y.; Murakami, A.; Fujimura, Y.; Tachibana, H.; Yamada, K.; Masuda, D.; Hirano, K.-i.; Yamashita, S.; Ohigashi, H. Aggregated ursolic acid, a natural triterpenoid, induces IL-1 beta release from murine peritoneal macrophages: Role of CD36. JOURNAL OF IMMUNOLOGY 2007, 178, 4854-4864, doi:10.4049/jimmunol.178.8.4854.

21. Samivel, R.; Nagarajan, R.P.; Subramanian, U.; Khan, A.A.; Masmali, A.; Almubrad, T.; Akhtar, S. Research Article Inhibitory Effect of Ursolic Acid on Ultraviolet B Radiation-Induced Oxidative Stress and Proinflammatory Response-Mediated Senescence in Human Skin Dermal Fibroblasts. 2020.

22. Tsai, S.-J.; Yin, M.C. Antioxidative and anti-inflammatory protection of oleanolic acid and ursolic acid in PC12 cells. Journal of food science 2008, 73, H174-H178, doi:10.1111/j.1750-3841.2008.00864.x.

23. Checker, R.; Sandur, S.K.; Sharma, D.; Patwardhan, R.S.; Jayakumar, S.; Kohli, V.; Sethi, G.; Aggarwal, B.B.; Sainis, K.B.J.P.o. Potent anti-inflammatory activity of ursolic acid, a triterpenoid antioxidant, is mediated through suppression of NF-κB, AP-1 and NF-AT. 2012, 7, e31318.

24. Chen, J.; He, D.; Li, Q.; Wu, Z.; Huang, W.; Zhu, Y.J.I.J.C.E.M. Ursolic acid protects MC3T3-E1 cells against dexamethasone-mediated apoptosis, ROS generation and inflammation through activation of IGF-1. 2016, 9, 23019-23030.

25. Chun, J.; Lee, C.; Hwang, S.W.; Im, J.P.; Kim, J.S. Ursolic acid inhibits nuclear factor-kappa B signaling in intestinal epithelial cells and macrophages, and attenuates experimental colitis in mice. Life sciences 2014, 110, 23-34, doi:10.1016/j.lfs.2014.06.018.

26. Lee, C.-H.; Wu, S.-L.; Chen, J.-C.; Li, C.-C.; Lo, H.-Y.; Cheng, W.-Y.; Lin, J.-G.; Chang, Y.-H.; Hsiang, C.-Y.; Ho, T.-Y. Eriobotrya japonica Leaf and Its Triterpenes Inhibited Lipopolysaccharide-Induced Cytokines and Inducible Enzyme Production via the Nuclear Factor-kappa B Signaling Pathway in Lung Epithelial Cells. AMERICAN JOURNAL OF CHINESE MEDICINE 2008, 36, 1185-1198, doi:10.1142/S0192415X0800651X.

27. Manu, K.A.; Kuttan, G. Ursolic acid induces apoptosis by activating p53 and caspase-3 gene expressions and suppressing NF-kappa B mediated activation of bcl-2 in B16F-10 melanoma cells. International immunopharmacology 2008, 8, 974-981, doi:10.1016/j.intimp.2008.02.013.

28. Moon, P.-D.; Han, N.-R.; Lee, J.S.; Kim, H.-M.; Jeong, H.-J. Ursolic acid downregulates thymic stromal lymphopoietin through the blockade of intracellular calcium/caspase-1/NF-kappa B signaling cascade in HMC-1 cells. International journal of molecular medicine 2019, 43, 2252-2258, doi:10.3892/ijmm.2019.4144.

29. Wang, H.; Sim, M.-K.; Loke, W.K.; Chinnathambi, A.; Alharbi, S.A.; Tang, F.R.; Sethi, G. Potential Protective Effects of Ursolic Acid against Gamma Irradiation-Induced Damage Are Mediated through the Modulation of Diverse Inflammatory Mediators. Frontiers in pharmacology 2017, 8, doi:10.3389/fphar.2017.00352.

30. Wei, X.; Lan, Y.; Nong, Z.; Li, C.; Feng, Z.; Mei, X.; Zhai, Y.; Zou, M.J.C. Ursolic acid represses influenza A virus-triggered inflammation and oxidative stress in A549 cells by modulating the miR-34c-5p/TLR5 axis. 2022, 157, 155947.

31. Yu, H.-H.; Hur, J.-M.; Seo, S.-J.; Moon, H.-D.; Kim, H.-J.; Park, R.-K.; You, Y.-O. Protective Effect of Ursolic Acid from Cornus officinalis on the Hydrogen Peroxide-Induced Damage of HEI-OC1 Auditory Cells. AMERICAN JOURNAL OF CHINESE MEDICINE 2009, 37, 735-746, doi:10.1142/S0192415X0900720X.
